# 6,6′-[(1*E*,1′*E*)-Oxybis(4,1-phenyl­ene)bis­(aza­nylyl­idene)bis­(methanylyl­idene)]bis­(2-methyl­phenol): supra­molecular assemblies in two dimensions mediated by weak C—H⋯N, C—H⋯O and C—H⋯π inter­actions

**DOI:** 10.1107/S2056989018005959

**Published:** 2018-04-24

**Authors:** Md. Azharul Arafath, Huey Chong Kwong, Farook Adam, Mohd. R. Razali

**Affiliations:** a School of Chemical Sciences, Universiti Sains Malaysia, Penang 11800 USM, Malaysia; b Department of Chemistry, Shahjalal University of Science and Technology, Sylhet 3114, Bangladesh

**Keywords:** crystal structure, oxybis Schiff base, T= 100 K, inter­molecular inter­action

## Abstract

The title compound, C_28_H_24_N_2_O_3_, is a flexible Schiff base, having a dihedral angle of 59.53 (5)° between the mean planes of two phenyl rings bounded in the centre by a single O atom. The dihedral angles between the mean planes of the phenyl rings bonded to the central O atom and the mean planes of the terminal methyl­phenol rings are 31.47 (6) and 36.03 (5)°, respectively. The *sp* ^2^-hybridized character of the azanylylidene groups is confirmed by their bond lengths and bond angles. In the crystal, mol­ecules are linked into centrosymmetric dimers by weak C—H⋯N inter­actions and connected into dimeric chains through weak C—H⋯O inter­actions. These chains are inter­connected into a two-dimensional network parallel to (11) *via* weak C—H⋯π inter­actions.

## Abstract 2

The title compound is a flexible Schiff base, as illustrated by its dihedral angles. The *sp* ^2^-hybridized character of the aza­nylyl­idene groups is confirmed by their bond lengths and bond angles. In the crystal, mol­ecules of the title compound are assembled into two-dimensional networks connected by weak C—H⋯O, C—H⋯N and C—H⋯π inter­molecular inter­actions.

## Chemical context   

The oxybis Schiff base compound is an important group in chemistry. Bis-carbazones are formed by connecting *via* a ring or C—C bond to carbazone moieties having four coordinated sites. These tetra­dentate ligands can be used to entrap metal ions to form square-planer complexes (Alsop *et al.*, 2005 ▸; Blower *et al.*, 2003 ▸; Jasinski *et al.*, 2003 ▸). The length of the C—C bond in the backbone of the compounds affects the stability of the complexes. The higher the number of C—C bonds (obtained *via* alkyl­ation or aryl­ation) allows the cavity within the ligand to fit the metal ion with a proper orientation (Blower *et al.*, 2003 ▸). These tetra­dentate compounds and transition metal complexes have potential anti­cancer and anti­bacterial activity (Lobana *et al.*, 2009 ▸). The bis compounds chelate to transition metal ions *via* coordination sites to form complexes that may also exhibit fluorescent properties that could be used as biosensors and chemosensors (Liu *et al.*, 2011 ▸; Jiang & Guo, 2004 ▸).
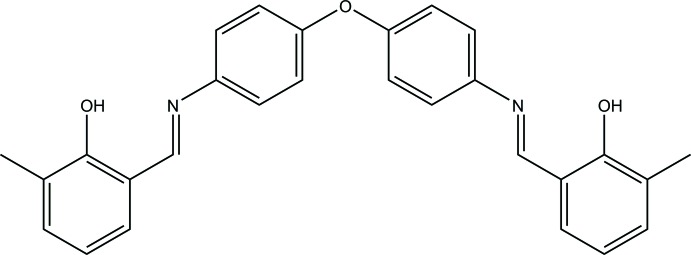


In view of the above mentioned properties and of our research inter­est in the synthesis of oxybis Schiff base compounds, we present in this study the crystal structure and supra­molecular features of the flexible Schiff base ligand 6,6′-{(1*E*,1′*E*)-[oxybis(4,1-phenyl­ene)bis­(aza­nyl­yl­idene)bis­(methan­ylyl­idene)]bis­(2-methyl­phenol}.

## Structural commentary   

**Figure 1:**
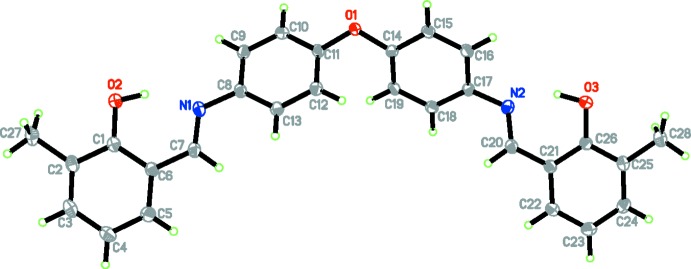
The title mol­ecule with the atom-labelling scheme and 50% probability displacement ellipsoids.

In the title oxybisbenzenyl compound (Fig. 1 ▸), the mean planes of the phenyl rings bonded to the central oxygen atom form a dihedral angle of 59.53 (5)°, and the mean planes of the C1–C6 and C21–C26 methyl­phenol rings are twisted similarly by 31.47 (6) and 36.03 (5)°, respectively, from the adjacent phenyl rings. The terminal methyl­phenol rings are almost parallel to each other, forming a dihedral angle of 2.46 (6)° between their mean planes. The C7=N1 and C20=N2 bond lengths of 1.2880 (14) Å and 1.2834 (13) Å, confirm the presence of the double bonds while the C8—N1 and C17—N2 bond lengths, 1.4156 (12) and 1.4154 (12) Å, respectively, confirm their single-bond character. The C7—N1—C8, C17—N2—C20, N1—C7—C6 and N2—C20—C21 angles are 121.11 (9), 119.51 (9), 121.63 (9) and 122.42 (9)°, respectively. These values are consistent with a *sp* ^2^-hybridized character for atoms C7, C20, N1 and N2 (Khalaji *et al.*, 2012 ▸). Two intra­molecular N—H⋯O hydrogen bonds occur (Table 1 ▸).

## Supra­molecular features   

**Figure 2:**
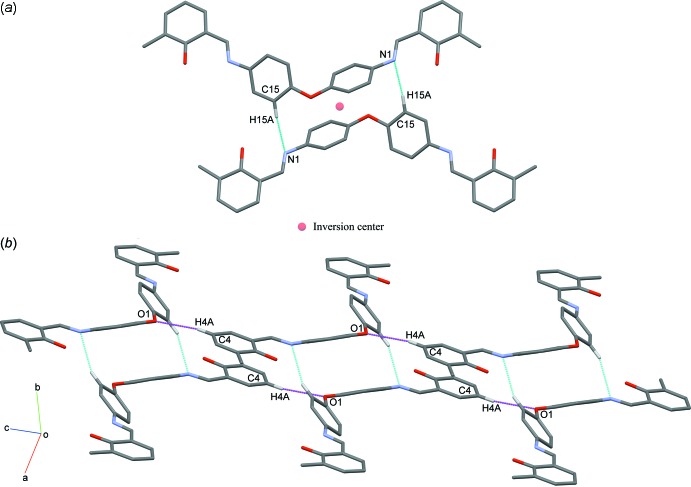
(*a*) A view of a centrosymmetric dimer of C_28_H_24_N_2_O_3_ with weak inter­molecular C15—H15*A*⋯N1 inter­actions shown as cyan dotted lines. (*b*) A view of a dimeric chain with weak inter­molecular C4—H4*A*⋯O1 shown as megenta lines. Hydrogen atoms not involved in with these inter­actions are omitted for clarity.

**Figure 3:**
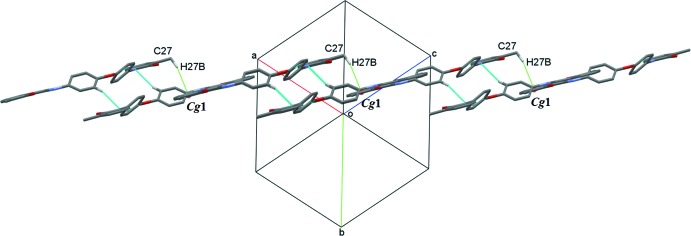
A view along (111) showing weak C—H⋯π (green dotted lines) supra­molecular inter­actions in the title compound.

In the crystal, mol­ecules are linked into centrosymmetric dimers by weak C15—H15*A*⋯N1 inter­actions forming an 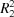
(18) ring motif (Fig. 2 ▸ *a*, Table 1 ▸). These dimers are linked into chains propagating along [111] by weak C4—H4*A*⋯O1 inter­actions (Fig. 2 ▸ *b*). At the same time, these dimeric chains are further connected into a two-dimensional network parallel to (121) *via* C—H⋯π inter­actions (Fig. 3 ▸, Table 1 ▸).

## Synthesis and crystallization   

**Figure 4:**
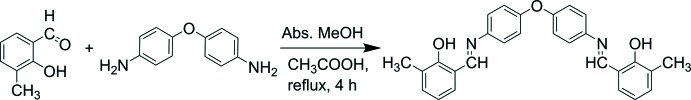
Reaction scheme for the synthesis of the title compound.

To a sample of 2-hy­droxy-3-methyl­benzaldehyde (0.68 g, 5.00 mmol) dissolved in 20.0 ml methanol was added 0.20 ml glacial acetic acid and the mixture was refluxed for 30 min. A solution of 4,4′-oxydianiline (0.50 g, 2.50 mmol) in 20.0 ml methanol was then added dropwise with stirring to the aldehyde solution. The resulting yellow solution was refluxed for 4 h (Fig. 4 ▸). A yellow-coloured precipitate formed. The precipitate was filtered and washed with 5.0 ml ethanol and 5.0 ml *n*-hexane. The recovered product was dissolved in acetone for recrystallization. Yellow single crystals suitable for X-ray diffraction were obtained by slow evaporation of acetone.

**6,6′-{(1** ***E*** **,1′** ***E*** **)-[Oxybis(4,1-phenyl­ene)bis­(aza­nylyl­idene)bis­(methanylyl­idene)]bis­(2-methyl­phenol}:** m.p. 398–399 K; yield 96%. IR (KBr pellets υ_max_/cm^−1^): 3430 υ(OH), 2884 υ(CH_3_), 1612 υ(C=N), 1496 υ(C=C, aromatic), 1272 υ(C–H, aromatic), 1239 υ(C—O, ether), 1195 υ(C—O, phenol), 1081 υ(C—N). ^1^H NMR (500 MHz, DMSO-*d*_6_, Me_4_Si ppm): δ 13.581 [*s* (1.97 H), OH], δ 8.952 [*s* (2.00 H), HC=N], δ 7.504–6.888 [multiplet (13.86 H), aromatic], δ 2.221 [*s* (6.11 H), Ph—CH_3_H ppm. The ^13^C NMR (DMSO-*d*_6_, Me4Si ppm): δ 163.21 (C=N), δ 158.60–118.32 (C-aromatic), δ 15.13 (CH_3_) ppm. Analysis calculated for C_28_H_24_N_2_O_3_ (FW: 436.51 g mol^−1^) C, 77.00; H, 5.50; N, 6.42; found: C, 77.05; H, 5.48; N, 6.40%.

## Refinement   

Crystal data, data collection and structure refinement details are summarized in Table 2 ▸. The phenolic hydrogen atoms were located in difference-Fourier maps and refined freely. All other H atoms attached calculated geometrically and refined using a riding model with C—H = 0.95–0.98 Å and *U*_iso_(H) = 1.2*U*_eq_(C) or 1.5*U*_eq_(C-meth­yl).

## Supplementary Material

Crystal structure: contains datablock(s) I. DOI: 10.1107/S2056989018005959/jj2197sup1.cif

Structure factors: contains datablock(s) I. DOI: 10.1107/S2056989018005959/jj2197Isup2.hkl

Supporting information file. DOI: 10.1107/S2056989018005959/jj2197Isup3.cml

CCDC reference: 1435817

Additional supporting information:  crystallographic information; 3D view; checkCIF report

## Figures and Tables

**Table 1:** Hydrogen-bond geometry (Å, °) *Cg*1 is the centroid of the C14–C19 ring.

*D*—H⋯*A*	*D*—H	H⋯*A*	*D*⋯*A*	*D*—H⋯*A*
O3—H1*O*3⋯N2	0.99 (2)	1.73 (2)	2.6441 (13)	151.0 (17)
O2—H1*O*2⋯N1	0.91 (2)	1.76 (2)	2.6011 (13)	151.4 (18)
C15—H15*A*⋯N1^i^	0.95	2.53	3.4211 (15)	156
C4—H4*A*⋯O1^ii^	0.95	2.72	3.6626 (14)	171
C27—H27*A*⋯*Cg*1^iii^	0.98	2.98	3.9242 (14)	162

Symmetry codes: (i) 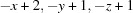; (ii) 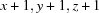; (iii) 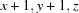.

**Table 2:** Experimental details

Crystal data	
Chemical formula	C_28_H_24_N_2_O_3_
*M*_r_	436.49
Crystal system, space group	Triclinic, *P* 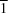
Temperature (K)	100
*a*, *b*, *c* (Å)	10.2293 (4), 10.9623 (4), 11.3087 (4)
α, β, γ (°)	108.5568 (10), 96.7616 (10), 110.4087 (10)
*V* (Å^3^)	1088.76 (7)
*Z*	2
Radiation type	Mo *K*α
μ (mm^−1^)	0.09
Crystal size (mm)	0.35 × 0.31 × 0.13

Data collection	
Diffractometer	Bruker APEXII DUO CCD area-detector
Absorption correction	Multi-scan (*SADABS*; Bruker, 2012 ▸)
*T*_min_, *T*_max_	0.903, 0.960
No. of measured, independent and observed [*I* > 2σ(*I*)] reflections	42726, 6513, 5433
*R*_int_	0.029
(sin θ/λ)_max_ (Å^−1^)	0.711

Refinement	
*R*[*F* ^2^ > 2σ(*F* ^2^)], *wR*(*F* ^2^), *S*	0.045, 0.131, 1.03
No. of reflections	6513
No. of parameters	308
H-atom treatment	H atoms treated by a mixture of independent and constrained refinement
Δρ_max_, Δρ_min_ (e Å^−3^)	0.38, −0.32

Computer programs: *APEX2* and *SAINT* (Bruker, 2012 ▸), *SHELXS97* (Sheldrick, 2008 ▸), *SHELXL2013* (Sheldrick, 2015 ▸), *Mercury* (Macrae *et al.*, 2006 ▸) and *PLATON* (Spek, 2009 ▸).

## supplementary crystallographic information

### Crystal data

**Table:**

C_28_H_24_N_2_O_3_	*Z* = 2
*M**_r_* = 436.49	*F*(000) = 460
Triclinic, *P*1	*D*_x_ = 1.331 Mg m^−^^3^
*a* = 10.2293 (4) Å	Mo *K*α radiation, λ = 0.71073 Å
*b* = 10.9623 (4) Å	Cell parameters from 9984 reflections
*c* = 11.3087 (4) Å	θ = 2.2–30.2°
α = 108.5568 (10)°	µ = 0.09 mm^−^^1^
β = 96.7616 (10)°	*T* = 100 K
γ = 110.4087 (10)°	Block, yellow
*V* = 1088.76 (7) Å^3^	0.35 × 0.31 × 0.13 mm

### Data collection

**Table:**

Bruker APEXII DUO CCD area-detector diffractometer	6513 independent reflections
Radiation source: fine-focus sealed tube	5433 reflections with *I* > 2σ(*I*)
Graphite monochromator	*R*_int_ = 0.029
φ and ω scans	θ_max_ = 30.4°, θ_min_ = 2.0°
Absorption correction: multi-scan (SADABS; Bruker, 2012)	*h* = −14→14
*T*_min_ = 0.903, *T*_max_ = 0.960	*k* = −15→15
42726 measured reflections	*l* = −16→15

### Refinement

**Table:**

Refinement on *F*^2^	0 restraints
Least-squares matrix: full	Hydrogen site location: mixed
*R*[*F*^2^ > 2σ(*F*^2^)] = 0.045	H atoms treated by a mixture of independent and constrained refinement
*wR*(*F*^2^) = 0.131	*w* = 1/[σ^2^(*F*_o_^2^) + (0.0747*P*)^2^ + 0.3053*P*] where *P* = (*F*_o_^2^ + 2*F*_c_^2^)/3
*S* = 1.03	(Δ/σ)_max_ = 0.001
6513 reflections	Δρ_max_ = 0.38 e Å^−^^3^
308 parameters	Δρ_min_ = −0.32 e Å^−^^3^

### Special details

**Table:**

Experimental. The following wavelength and cell were deduced by SADABS from the direction cosines etc. They are given here for emergency use only: CELL 0.71062 10.322 11.055 11.397 108.521 96.732 110.436
Geometry. All esds (except the esd in the dihedral angle between two l.s. planes) are estimated using the full covariance matrix. The cell esds are taken into account individually in the estimation of esds in distances, angles and torsion angles; correlations between esds in cell parameters are only used when they are defined by crystal symmetry. An approximate (isotropic) treatment of cell esds is used for estimating esds involving l.s. planes.

### Fractional atomic coordinates and isotropic or equivalent isotropic displacement parameters (Å^2^)

**Table:**

	*x*	*y*	*z*	*U*_iso_*/*U*_eq_	
O1	0.76527 (8)	0.48598 (7)	0.45322 (7)	0.01951 (16)	
O2	1.54997 (8)	1.09474 (8)	0.70552 (7)	0.02197 (16)	
O3	0.06378 (8)	0.07922 (8)	0.64561 (7)	0.02142 (16)	
N1	1.29823 (9)	0.93739 (9)	0.71785 (9)	0.01841 (17)	
N2	0.32630 (9)	0.26192 (9)	0.67203 (8)	0.01845 (17)	
C1	1.57792 (11)	1.16561 (10)	0.83396 (10)	0.01812 (19)	
C2	1.70943 (11)	1.28524 (11)	0.89546 (11)	0.0208 (2)	
C3	1.73960 (11)	1.35727 (11)	1.02776 (11)	0.0237 (2)	
H3A	1.8280	1.4382	1.0703	0.028*	
C4	1.64482 (12)	1.31511 (11)	1.10050 (11)	0.0237 (2)	
H4A	1.6693	1.3654	1.1912	0.028*	
C5	1.51434 (11)	1.19879 (11)	1.03847 (10)	0.0218 (2)	
H5A	1.4485	1.1701	1.0870	0.026*	
C6	1.47866 (11)	1.12316 (10)	0.90474 (10)	0.01809 (19)	
C7	1.33802 (11)	1.00598 (11)	0.84107 (10)	0.01925 (19)	
H7A	1.2739	0.9794	0.8916	0.023*	
C8	1.16198 (10)	0.82216 (10)	0.65723 (10)	0.01726 (19)	
C9	1.09958 (11)	0.79317 (10)	0.52934 (10)	0.01908 (19)	
H9A	1.1491	0.8497	0.4867	0.023*	
C10	0.96575 (11)	0.68236 (10)	0.46405 (10)	0.01876 (19)	
H10A	0.9225	0.6645	0.3778	0.023*	
C11	0.89561 (10)	0.59785 (10)	0.52567 (9)	0.01665 (18)	
C12	0.95985 (10)	0.62060 (10)	0.65062 (10)	0.01793 (19)	
H12A	0.9136	0.5592	0.6905	0.022*	
C13	1.09192 (10)	0.73371 (10)	0.71658 (10)	0.01777 (19)	
H13A	1.1350	0.7511	0.8028	0.021*	
C14	0.66246 (10)	0.43684 (10)	0.51590 (9)	0.01645 (18)	
C15	0.57873 (10)	0.29247 (10)	0.46457 (10)	0.01831 (19)	
H15A	0.5973	0.2321	0.3937	0.022*	
C16	0.46798 (11)	0.23692 (10)	0.51734 (10)	0.01851 (19)	
H16A	0.4099	0.1383	0.4817	0.022*	
C17	0.44103 (10)	0.32475 (10)	0.62243 (9)	0.01667 (18)	
C18	0.52465 (10)	0.47018 (10)	0.67105 (9)	0.01735 (19)	
H18A	0.5059	0.5310	0.7414	0.021*	
C19	0.63458 (10)	0.52671 (10)	0.61781 (9)	0.01706 (19)	
H19A	0.6902	0.6257	0.6506	0.020*	
C20	0.33650 (11)	0.31399 (11)	0.79342 (10)	0.01902 (19)	
H20A	0.4225	0.3926	0.8481	0.023*	
C21	0.22143 (10)	0.25721 (10)	0.85028 (9)	0.01744 (19)	
C22	0.24188 (11)	0.31750 (11)	0.98398 (10)	0.0217 (2)	
H22A	0.3289	0.3969	1.0353	0.026*	
C23	0.13736 (12)	0.26316 (12)	1.04218 (11)	0.0247 (2)	
H23A	0.1518	0.3047	1.1329	0.030*	
C24	0.01010 (12)	0.14628 (12)	0.96590 (11)	0.0237 (2)	
H24A	−0.0613	0.1085	1.0064	0.028*	
C25	−0.01556 (11)	0.08364 (11)	0.83349 (10)	0.0201 (2)	
C26	0.09098 (11)	0.14035 (10)	0.77475 (9)	0.01703 (18)	
C27	1.81029 (12)	1.33195 (13)	0.81651 (12)	0.0279 (2)	
H27A	1.7559	1.3335	0.7401	0.042*	
H27B	1.8558	1.2660	0.7894	0.042*	
H27C	1.8849	1.4267	0.8686	0.042*	
C28	−0.15322 (12)	−0.04094 (12)	0.75086 (12)	0.0289 (2)	
H28A	−0.1306	−0.1120	0.6897	0.043*	
H28B	−0.2132	−0.0103	0.7032	0.043*	
H28C	−0.2056	−0.0817	0.8058	0.043*	
H1O3	0.153 (2)	0.131 (2)	0.6250 (18)	0.052 (5)*	
H1O2	1.458 (2)	1.027 (2)	0.6813 (19)	0.056 (5)*	

### Atomic displacement parameters (Å^2^)

**Table:**

	*U*^11^	*U*^22^	*U*^33^	*U*^12^	*U*^13^	*U*^23^
O1	0.0152 (3)	0.0190 (3)	0.0165 (3)	0.0016 (3)	0.0049 (3)	0.0028 (3)
O2	0.0198 (4)	0.0216 (4)	0.0199 (4)	0.0045 (3)	0.0050 (3)	0.0068 (3)
O3	0.0221 (4)	0.0196 (3)	0.0162 (3)	0.0042 (3)	0.0033 (3)	0.0043 (3)
N1	0.0148 (4)	0.0158 (4)	0.0220 (4)	0.0048 (3)	0.0036 (3)	0.0060 (3)
N2	0.0165 (4)	0.0193 (4)	0.0205 (4)	0.0071 (3)	0.0064 (3)	0.0086 (3)
C1	0.0165 (4)	0.0169 (4)	0.0213 (5)	0.0074 (4)	0.0037 (4)	0.0075 (4)
C2	0.0165 (4)	0.0184 (4)	0.0269 (5)	0.0066 (4)	0.0030 (4)	0.0097 (4)
C3	0.0169 (4)	0.0191 (5)	0.0290 (5)	0.0056 (4)	−0.0009 (4)	0.0060 (4)
C4	0.0219 (5)	0.0230 (5)	0.0216 (5)	0.0105 (4)	0.0006 (4)	0.0032 (4)
C5	0.0197 (5)	0.0234 (5)	0.0217 (5)	0.0104 (4)	0.0045 (4)	0.0066 (4)
C6	0.0162 (4)	0.0169 (4)	0.0203 (5)	0.0069 (3)	0.0039 (4)	0.0062 (4)
C7	0.0153 (4)	0.0178 (4)	0.0239 (5)	0.0061 (4)	0.0059 (4)	0.0075 (4)
C8	0.0139 (4)	0.0155 (4)	0.0208 (5)	0.0056 (3)	0.0048 (3)	0.0053 (4)
C9	0.0192 (4)	0.0176 (4)	0.0200 (5)	0.0062 (4)	0.0068 (4)	0.0076 (4)
C10	0.0192 (4)	0.0191 (4)	0.0169 (4)	0.0075 (4)	0.0047 (4)	0.0060 (4)
C11	0.0137 (4)	0.0152 (4)	0.0180 (4)	0.0052 (3)	0.0043 (3)	0.0032 (3)
C12	0.0165 (4)	0.0178 (4)	0.0199 (5)	0.0067 (4)	0.0057 (4)	0.0077 (4)
C13	0.0163 (4)	0.0190 (4)	0.0183 (4)	0.0076 (4)	0.0042 (3)	0.0071 (4)
C14	0.0135 (4)	0.0183 (4)	0.0158 (4)	0.0053 (3)	0.0038 (3)	0.0057 (3)
C15	0.0175 (4)	0.0171 (4)	0.0171 (4)	0.0067 (4)	0.0042 (3)	0.0032 (4)
C16	0.0171 (4)	0.0156 (4)	0.0192 (4)	0.0046 (3)	0.0035 (3)	0.0048 (4)
C17	0.0136 (4)	0.0185 (4)	0.0165 (4)	0.0056 (3)	0.0031 (3)	0.0064 (3)
C18	0.0165 (4)	0.0175 (4)	0.0166 (4)	0.0069 (3)	0.0037 (3)	0.0049 (3)
C19	0.0151 (4)	0.0150 (4)	0.0172 (4)	0.0045 (3)	0.0027 (3)	0.0039 (3)
C20	0.0154 (4)	0.0183 (4)	0.0207 (5)	0.0052 (3)	0.0035 (3)	0.0066 (4)
C21	0.0166 (4)	0.0173 (4)	0.0173 (4)	0.0064 (3)	0.0039 (3)	0.0060 (4)
C22	0.0203 (5)	0.0204 (5)	0.0190 (5)	0.0053 (4)	0.0030 (4)	0.0048 (4)
C23	0.0264 (5)	0.0266 (5)	0.0183 (5)	0.0094 (4)	0.0069 (4)	0.0064 (4)
C24	0.0226 (5)	0.0252 (5)	0.0245 (5)	0.0083 (4)	0.0104 (4)	0.0113 (4)
C25	0.0177 (4)	0.0179 (4)	0.0228 (5)	0.0054 (4)	0.0046 (4)	0.0078 (4)
C26	0.0174 (4)	0.0156 (4)	0.0176 (4)	0.0071 (3)	0.0035 (3)	0.0058 (3)
C27	0.0200 (5)	0.0257 (5)	0.0348 (6)	0.0039 (4)	0.0055 (4)	0.0142 (5)
C28	0.0216 (5)	0.0248 (5)	0.0303 (6)	0.0009 (4)	0.0042 (4)	0.0089 (5)

### Geometric parameters (Å, º)

**Table:**

O1—C11	1.3841 (11)	C12—H12A	0.9500
O1—C14	1.3864 (11)	C13—H13A	0.9500
O2—C1	1.3506 (13)	C14—C15	1.3885 (13)
O2—H1O2	0.91 (2)	C14—C19	1.3895 (13)
O3—C26	1.3456 (12)	C15—C16	1.3855 (14)
O3—H1O3	0.988 (19)	C15—H15A	0.9500
N1—C7	1.2880 (14)	C16—C17	1.3962 (14)
N1—C8	1.4156 (12)	C16—H16A	0.9500
N2—C20	1.2834 (13)	C17—C18	1.3985 (13)
N2—C17	1.4154 (12)	C18—C19	1.3864 (13)
C1—C2	1.4078 (14)	C18—H18A	0.9500
C1—C6	1.4097 (14)	C19—H19A	0.9500
C2—C3	1.3880 (16)	C20—C21	1.4521 (14)
C2—C27	1.5045 (15)	C20—H20A	0.9500
C3—C4	1.3956 (16)	C21—C22	1.3998 (14)
C3—H3A	0.9500	C21—C26	1.4122 (13)
C4—C5	1.3866 (15)	C22—C23	1.3792 (15)
C4—H4A	0.9500	C22—H22A	0.9500
C5—C6	1.4030 (14)	C23—C24	1.3962 (15)
C5—H5A	0.9500	C23—H23A	0.9500
C6—C7	1.4549 (14)	C24—C25	1.3819 (15)
C7—H7A	0.9500	C24—H24A	0.9500
C8—C13	1.3954 (14)	C25—C26	1.4058 (14)
C8—C9	1.3960 (14)	C25—C28	1.5027 (15)
C9—C10	1.3879 (14)	C27—H27A	0.9800
C9—H9A	0.9500	C27—H27B	0.9800
C10—C11	1.3870 (14)	C27—H27C	0.9800
C10—H10A	0.9500	C28—H28A	0.9800
C11—C12	1.3905 (14)	C28—H28B	0.9800
C12—C13	1.3874 (13)	C28—H28C	0.9800
			
C11—O1—C14	118.92 (7)	C16—C15—H15A	120.2
C1—O2—H1O2	105.6 (12)	C14—C15—H15A	120.2
C26—O3—H1O3	104.7 (11)	C15—C16—C17	120.52 (9)
C7—N1—C8	121.11 (9)	C15—C16—H16A	119.7
C20—N2—C17	119.51 (9)	C17—C16—H16A	119.7
O2—C1—C2	117.80 (9)	C16—C17—C18	118.95 (9)
O2—C1—C6	121.55 (9)	C16—C17—N2	117.94 (9)
C2—C1—C6	120.64 (9)	C18—C17—N2	123.08 (9)
C3—C2—C1	118.03 (10)	C19—C18—C17	120.84 (9)
C3—C2—C27	122.46 (10)	C19—C18—H18A	119.6
C1—C2—C27	119.49 (10)	C17—C18—H18A	119.6
C2—C3—C4	122.38 (10)	C18—C19—C14	119.21 (9)
C2—C3—H3A	118.8	C18—C19—H19A	120.4
C4—C3—H3A	118.8	C14—C19—H19A	120.4
C5—C4—C3	119.06 (10)	N2—C20—C21	122.42 (9)
C5—C4—H4A	120.5	N2—C20—H20A	118.8
C3—C4—H4A	120.5	C21—C20—H20A	118.8
C4—C5—C6	120.60 (10)	C22—C21—C26	119.10 (9)
C4—C5—H5A	119.7	C22—C21—C20	119.12 (9)
C6—C5—H5A	119.7	C26—C21—C20	121.75 (9)
C5—C6—C1	119.24 (9)	C23—C22—C21	120.85 (10)
C5—C6—C7	119.47 (9)	C23—C22—H22A	119.6
C1—C6—C7	121.25 (9)	C21—C22—H22A	119.6
N1—C7—C6	121.63 (9)	C22—C23—C24	119.09 (10)
N1—C7—H7A	119.2	C22—C23—H23A	120.5
C6—C7—H7A	119.2	C24—C23—H23A	120.5
C13—C8—C9	119.20 (9)	C25—C24—C23	122.23 (10)
C13—C8—N1	123.39 (9)	C25—C24—H24A	118.9
C9—C8—N1	117.33 (9)	C23—C24—H24A	118.9
C10—C9—C8	120.50 (9)	C24—C25—C26	118.34 (9)
C10—C9—H9A	119.7	C24—C25—C28	122.39 (10)
C8—C9—H9A	119.7	C26—C25—C28	119.27 (9)
C11—C10—C9	119.52 (9)	O3—C26—C25	117.66 (9)
C11—C10—H10A	120.2	O3—C26—C21	121.97 (9)
C9—C10—H10A	120.2	C25—C26—C21	120.37 (9)
O1—C11—C10	116.44 (9)	C2—C27—H27A	109.5
O1—C11—C12	122.74 (9)	C2—C27—H27B	109.5
C10—C11—C12	120.67 (9)	H27A—C27—H27B	109.5
C13—C12—C11	119.50 (9)	C2—C27—H27C	109.5
C13—C12—H12A	120.2	H27A—C27—H27C	109.5
C11—C12—H12A	120.2	H27B—C27—H27C	109.5
C12—C13—C8	120.47 (9)	C25—C28—H28A	109.5
C12—C13—H13A	119.8	C25—C28—H28B	109.5
C8—C13—H13A	119.8	H28A—C28—H28B	109.5
O1—C14—C15	116.46 (8)	C25—C28—H28C	109.5
O1—C14—C19	122.52 (9)	H28A—C28—H28C	109.5
C15—C14—C19	120.80 (9)	H28B—C28—H28C	109.5
C16—C15—C14	119.63 (9)		
			
O2—C1—C2—C3	−179.09 (9)	C11—O1—C14—C15	−144.71 (9)
C6—C1—C2—C3	1.68 (14)	C11—O1—C14—C19	40.71 (13)
O2—C1—C2—C27	2.00 (14)	O1—C14—C15—C16	−176.07 (8)
C6—C1—C2—C27	−177.23 (9)	C19—C14—C15—C16	−1.39 (15)
C1—C2—C3—C4	0.05 (16)	C14—C15—C16—C17	−0.68 (15)
C27—C2—C3—C4	178.92 (10)	C15—C16—C17—C18	1.97 (15)
C2—C3—C4—C5	−1.33 (16)	C15—C16—C17—N2	179.91 (9)
C3—C4—C5—C6	0.88 (16)	C20—N2—C17—C16	146.74 (10)
C4—C5—C6—C1	0.80 (15)	C20—N2—C17—C18	−35.40 (14)
C4—C5—C6—C7	−176.86 (9)	C16—C17—C18—C19	−1.23 (14)
O2—C1—C6—C5	178.69 (9)	N2—C17—C18—C19	−179.06 (9)
C2—C1—C6—C5	−2.11 (14)	C17—C18—C19—C14	−0.79 (14)
O2—C1—C6—C7	−3.69 (15)	O1—C14—C19—C18	176.47 (9)
C2—C1—C6—C7	175.51 (9)	C15—C14—C19—C18	2.12 (15)
C8—N1—C7—C6	179.01 (9)	C17—N2—C20—C21	178.32 (9)
C5—C6—C7—N1	177.01 (9)	N2—C20—C21—C22	178.44 (10)
C1—C6—C7—N1	−0.61 (15)	N2—C20—C21—C26	0.19 (15)
C7—N1—C8—C13	−30.61 (15)	C26—C21—C22—C23	0.78 (16)
C7—N1—C8—C9	152.59 (10)	C20—C21—C22—C23	−177.52 (10)
C13—C8—C9—C10	3.47 (15)	C21—C22—C23—C24	0.16 (17)
N1—C8—C9—C10	−179.59 (9)	C22—C23—C24—C25	−0.62 (17)
C8—C9—C10—C11	−1.67 (15)	C23—C24—C25—C26	0.10 (16)
C14—O1—C11—C10	−152.24 (9)	C23—C24—C25—C28	−179.15 (11)
C14—O1—C11—C12	32.17 (13)	C24—C25—C26—O3	−179.28 (9)
C9—C10—C11—O1	−177.53 (8)	C28—C25—C26—O3	0.00 (14)
C9—C10—C11—C12	−1.84 (15)	C24—C25—C26—C21	0.87 (15)
O1—C11—C12—C13	178.90 (9)	C28—C25—C26—C21	−179.86 (9)
C10—C11—C12—C13	3.49 (15)	C22—C21—C26—O3	178.85 (9)
C11—C12—C13—C8	−1.64 (15)	C20—C21—C26—O3	−2.90 (15)
C9—C8—C13—C12	−1.80 (15)	C22—C21—C26—C25	−1.30 (15)
N1—C8—C13—C12	−178.55 (9)	C20—C21—C26—C25	176.95 (9)

### Hydrogen-bond geometry (Å, º)

Cg1 is the centroid of the C14–C19 ring.

**Table:**

*D*—H···*A*	*D*—H	H···*A*	*D*···*A*	*D*—H···*A*
O3—H1*O*3···N2	0.99 (2)	1.73 (2)	2.6441 (13)	151.0 (17)
O2—H1*O*2···N1	0.91 (2)	1.76 (2)	2.6011 (13)	151.4 (18)
C15—H15*A*···N1^i^	0.95	2.53	3.4211 (15)	156
C4—H4*A*···O1^ii^	0.95	2.72	3.6626 (14)	171
C27—H27*A*···*Cg*1^iii^	0.98	2.98	3.9242 (14)	162

Symmetry codes: (i) −*x*+2, −*y*+1, −*z*+1; (ii) *x*+1, *y*+1, *z*+1; (iii) *x*+1, *y*+1, *z*.

## References

[bb1] Alsop, L., Cowley, A. R., Dilworth, J. R., Donnelly, P. S., Peach, J. M. & Rider, J. T. (2005). *Inorg. Chim. Acta*, **358**, 2770–2780.

[bb2] Blower, P. J., Castle, T. C., Cowley, A. R., Dilworth, J. R., Donnelly, P. S., Labisbal, E., Sowrey, F. E., Teat, S. J. & Went, M. J. (2003). *Dalton Trans.* pp. 4416–4425.

[bb3] Bruker (2012). *APEX2*, *SAINT* and *SADABS*. Bruker AXS Inc., Madison. Wisconsin, USA.

[bb4] Jasinski, J. P., Bianchani, J. R., Cueva, J., El-Saied, F. A., El-Asmy, A. A. & West, D. X. (2003). *Z. Anorg. Allg. Chem.* **629**, 202–206.

[bb5] Jiang, P. & Guo, Z. (2004). *Coord. Chem. Rev.* **248**, 205–229.

[bb6] Khalaji, A. D., Fejfarova, K. & Dusek, M. (2012). *J. Chem. Crystallogr.* **42**, 263–266.

[bb7] Liu, K., Shi, W. & Cheng, P. (2011). *Dalton Trans.* **40**, 8475–8490. 10.1039/c0dt01578d 21629962

[bb8] Lobana, T. S., Sharma, R., Bawa, G. & Khanna, S. (2009). *Coord. Chem. Rev.* **253**, 977–1055.

[bb9] Macrae, C. F., Edgington, P. R., McCabe, P., Pidcock, E., Shields, G. P., Taylor, R., Towler, M. & van de Streek, J. (2006). *J. Appl. Cryst.* **39**, 453–457.

[bb10] Sheldrick, G. M. (2008). *Acta Cryst.* A**64**, 112–122. 10.1107/S0108767307043930 18156677

[bb11] Sheldrick, G. M. (2015). *Acta Cryst.* C**71**, 3–8.

[bb12] Spek, A. L. (2009). *Acta Cryst.* D**65**, 148–155. 10.1107/S090744490804362X PMC2631630 19171970

